# Primary Open-Angle African American Glaucoma Genetics (POAAGG) Study: gender and risk of POAG in African Americans

**DOI:** 10.1371/journal.pone.0218804

**Published:** 2019-08-01

**Authors:** Naira Khachatryan, Maxwell Pistilli, Maureen G. Maguire, Rebecca J. Salowe, Raymond M. Fertig, Tanisha Moore, Harini V. Gudiseva, Venkata R. M. Chavali, David W. Collins, Ebenezer Daniel, Windell Murphy, Jeffrey D. Henderer, Amanda Lehman, Qi Cui, Victoria Addis, Prithvi S. Sankar, Eydie G. Miller-Ellis, Joan M. O’Brien

**Affiliations:** 1 Scheie Eye Institute, University of Pennsylvania, Philadelphia, PA, United States of America; 2 Independent Physician, Philadelphia, PA, United States of America; 3 Department of Ophthalmology, Lewis Katz School of Medicine at Temple University, Philadelphia, PA, United States of America; National Eye Institute, UNITED STATES

## Abstract

The purpose of this study was to investigate the association between gender and primary open-angle glaucoma (POAG) among African Americans and to assess demographic, systemic, and behavioral factors that may contribute to differences between genders. The Primary Open-Angle African American Glaucoma Genetics (POAAGG) study had a case-control design and included African Americans 35 years and older, recruited from the greater Philadelphia, Pennsylvania. Diagnosis of POAG was based on evidence of both glaucomatous optic nerve damage and characteristic visual field loss. Demographic and behavioral information, history of systemic diseases and anthropometric measurements were obtained at study enrollment. Gender differences in risk of POAG were examined using multivariate logistic regression. A total of 2,290 POAG cases and 2,538 controls were included in the study. The percentage of men among cases was higher than among controls (38.6% vs 30.3%, P<0.001). The subjects’ mean age at enrollment was significantly higher for cases compared to controls (70.2±11.3 vs. 61.6±11.8 years, P<0.003). Cases had lower rates of diabetes (40% vs. 46%, P<0.001), higher rates of systemic hypertension (80% vs. 72%, P<0.001), and lower body mass index (BMI) (29.7±6.7 vs. 31.9±7.4, P<0.001) than controls. In the final multivariable model, male gender was significantly associated with POAG risk (OR, 1.64; 95% CI, 1.44–1.87; P<0.001), after adjusting for age, systemic hypertension, diabetes, and BMI. Within the POAAGG study, men were at higher risk of having POAG than women. Pending genetic results from this study will be used to better understand the underlying genetic variations that may account for these differences.

## Introduction

Glaucoma is the leading cause of irreversible vision loss worldwide [1]. Primary open-angle glaucoma (POAG), the most common form of the disease, accounts for the majority (74%) of glaucoma cases [1]. In 2013, more than 44 million individuals were affected by POAG, with this number predicted to increase to between 53 million [2] and 58 million by 2020 [1]. This increased disease burden emphasizes the importance of identifying factors that affect POAG prevalence. Demographic variables, known as non-modifiable or inherent determinants of disease, can be important in identifying high-risk groups. Age is a strongly established risk factor for POAG, with prevalence in US adults increasing from 0.6% at ages 40–49 to 8.3% at age 80 or older [3]. Race is also a strong risk factor, with African Americans four to five times more likely to have glaucoma than European Americans [4]. However, there is little agreement on the role of gender in the development and severity of POAG.

Several population-based prevalence studies conducted in different regions of the world have reported inconsistent results regarding the prevalence of POAG between genders. The Baltimore Eye Survey [4], the Melbourne Visual Impairment Project [5], and the Projecto VER [6] showed no difference in prevalence by gender. In contrast, the Blue Mountain Eye Study revealed that the age-adjusted prevalence of glaucoma was higher in women, although this finding was of borderline significance [7]. However, the Barbados Eye Study [8], the Framingham Eye Study [9], the Rotterdam Study [10], and the Los Angeles Latino Eye Study [11] found a higher prevalence of glaucoma among men compared with women. These conflicting results may be due to varying definitions of glaucoma, selection of study sample, reporting bias, and other contributing factors such as racial composition of the study population.

The Primary Open-Angle African American Glaucoma Genetics (POAAGG) study is a five-year, case-control study funded by the National Eye Institute. The POAAGG study was established to identify the genetic risk factors that underlie POAG in the high-risk, understudied African American population [12]. This study has recruited the largest African American population with POAG to date [12]. Our report aims (i) to elucidate the relationship of gender and risk of POAG in this African American population and (ii) to investigate demographic, systemic, and behavioral factors that potentially contribute to gender disparities.

## Methods

### Study design and sample

The POAAGG study has a case-control design [12]. Potential study participants were identified from the University of Pennsylvania (UPenn), including the Scheie Eye Institute, Perelman Center for Advanced Medicine, Mercy Fitzgerald Hospital. Participants were also recruited from Temple University Hospital and a private practice in West Philadelphia (Windell Murphy, MD). A subset of patients was recruited from the Penn Medicine Biobank (PMBB).

POAAGG participants were age 35 years or older and self-identified as black (African American, African descent, or African Caribbean). Participants with coexisting history of ocular trauma, non-glaucomatous optic disc neuropathy, inflammatory eye diseases, Grave’s disease with ocular manifestations, pseudoexfoliation, or any type of glaucoma other than POAG were excluded.

UPenn-certified clinical research coordinators selected potential participants from the pool of subjects with appointments at the hospitals/eye clinics participating in the study (listed above). At enrollment, all subjects signed an informed consent form and provided a genomic DNA sample, which was extracted from peripheral blood or saliva. A standardized protocol was used for data collection, including a comprehensive interview and a series of measurements [12]. Examination data were recorded on case report forms and entered directly into the REDCap (Research Electronic Data Capture) database. In addition, retrospective ophthalmic and systemic data were extracted from the UPenn EPIC and MERGE databases containing information from clinical examinations.

PMBB provided the POAAGG study with data on African Americans 35 years and older, whose blood samples had previously been obtained. These patients had consented to have their health and genetic information used for research studies at UPenn. For these participants, ocular and systemic data were extracted from the UPenn EPIC and MERGE databases and data was requested from Penn Medicine's Clinical Data Warehouse (Penn Data Store). In addition, an ophthalmologist/postdoctoral researcher (NK) reviewed medical charts of PMBB participants with records at the UPenn EPIC and MERGE databases to extract ocular information. For the purposes of this report, only PMBB subjects with complete ophthalmic and systemic information of interest were included (n = 14).

The study conformed to the tenets of the Declaration of Helsinki and to the Health Insurance Portability and Accountability Act. The UPenn institutional review board approved the methods. Detailed information about the POAAGG study is provided elsewhere [12].

### Definition of case and controls

POAG cases were defined as having an open iridocorneal angle and: (1) characteristic glaucomatous optic nerve findings in one or both eyes consisting of at least one of the following: notching, neuroretinal rim thinning, excavation, or a nerve fiber layer defect; (2) characteristic visual field defects on two consecutive reliable visual field tests in at least one eye, which were consistent with the observed optic nerve defects in that eye, as determined by fellowship-trained glaucoma specialists; and (3) all secondary causes of glaucoma excluded.

Normal controls were defined as subjects without: (1) high myopia (greater than -8.00 diopters); (2) high hyperopia (+8.00 diopters); (3) abnormal visual field; (4) intraocular pressure (IOP) greater than 21 mmHg; (5) neuroretinal rim thinning, excavation, notching or nerve fiber layer defects; (6) optic nerve asymmetry; or (7) a cup to disc ratio difference between eyes greater than 0.2. For the purpose of this report from the POAAGG study, only controls with at least one full comprehensive eye examination conducted at a hospital/eye clinic participating in the POAAGG study were included.

### Socio-demographic information

Standardized interviews were conducted by coordinators at enrollment to obtain demographic, behavioral, and systemic disease information. Demographic information included age at enrollment, the most current information on participant's gender, and self-described race. Behavioral information, specifically smoking information, was collected using a number of questions. However, for the purpose of this report, we created an “umbrella” variable (“Smoking Status”) that included subcategories Never Smoker, Ever Smoker (past or current smoker), and Unknown.

### Clinical history

History of systemic diseases and anthropometric measurements were obtained both at the enrollment interview and from medical charts. During the interview, each participant was asked if he/she had been diagnosed with high blood pressure (hypertension). If the answer was yes, the participant was asked about medications used to control hypertension. If a participant confirmed that he/she used medications, but did not recall the name of medication, this information was obtained from medical chart review. The same methodology was used to collect information on diabetes status.

Weight and height information was obtained during the interview. If a participant didn’t recall her/his most recent measurements, this information was extracted from medical charts. Body mass index (BMI) was calculated by dividing weight (lb.) by height (in.) squared and multiplying by a conversion factor of 703. BMI included the following categories: (i) Normal or Underweight (BMI less than 25), (ii) Overweight (BMI of 25 or more but less than 30), or (iii) Obese (BMI of 30 or more).

### Data management and analysis

We examined the unadjusted association between POAG and gender, age, diabetes, hypertension, body mass index, and smoking status all other characteristics using logistic regression. Multivariable logistic regression models were used to adjust for imbalances between cases and controls on risk factors other than gender. Only those variables associated with significant two-sided p-values in the unadjusted analysis were included in the final model. The interaction between age and gender was tested to assess the homogeneity of the odds ratio between gender and POAG. Statistical analysis was performed using SAS software version 9.4 (SAS Inc., Cary, NC).

## Results

Overall, 4,828 participants were included in this study, including 2,290 cases and 2,538 controls. The proportion of men among cases was significantly higher than among controls (38.6% vs 30.3%, OR 1.45, 95% CI, 1.29–1.89, P<0.001) (Table 1).

**Table 1:** The Primary Open-Angle African American Glaucoma Genetics (POAAGG) Study: Demographics, systemic and behavioral characteristics for study participants.

Characteristics at enrollment		Total n = 4828	Controls	Cases	Cases vs Controls	p-value
			n = 2538 (53%)	n = 2290 (47%)	Odds Ratio (95% CI)	
**Gender**	**Female**	3175 (66%)	1770 (70%)	1405 (61%)	1	<0.001
	**Male**	1653 (34%)	768 (30%)	885 (39%)	1.45 (1.29, 1.64)	
**Age, years (per 10)**	**Mean (Standard Deviation)**	65.7 (12.3)	61.6 (11.8)	70.2 (11.3)	1.89 (1.79, 2.00)	<0.001
**Age groups, years**	**<50**	516 (11%)	431 (17%)	85 (4%)	1	<0.001
	**50-<60**	1016 (21%)	682 (27%)	334 (15%)	2.48 (1.90, 3.24)	
	**60-<70**	1390 (29%)	762 (30%)	628 (27%)	4.18 (3.24, 5.40)	
	**70-<80**	1220 (25%)	486 (19%)	734 (32%)	7.66 (5.91, 9.93)	
	**>80**	686 (14%)	177 (7%)	509 (22%)	14.58 (10.92, 19.46)	
**Diabetes**	**No**	2732 (57%)	1367 (54%)	1365 (60%)	1	<0.001
	**Yes**	2096 (43%)	1171 (46%)	925 (40%)	0.79 (0.71, 0.89)	
**Hypertension**	**No**	1169 (24%)	714 (28%)	455 (20%)	1	<0.001
	**Yes**	3659 (76%)	1824 (72%)	1835 (80%)	1.58 (1.38, 1.81)	
**Body Mass Index (BMI), kg/m**^**2**^ **(per 5)**	**Mean (Standard Deviation)**	30.9 (7.1)	31.9 (7.4)	29.7 (6.7)	0.80 (0.76, 0.83)	<0.001
**BMI categories, kg/m**^**2**^	**Normal or Underweight (<25)**	899 (19%)	376 (15%)	523 (23%)	1	<0.001
	**Overweight (25-<30)**	1556 (32%)	761 (30%)	795 (35%)	0.75 (0.64, 0.89)	
	**Obese (> = 30)**	2373 (49%)	1401 (55%)	972 (42%)	0.50 (0.43, 0.58)	
**Smoking Status**	**Never Smoker**	2138 (44%)	1138 (45%)	1000 (44%)	1	0.71
	**Ever Smoker**	2496 (52%)	1298 (51%)	1198 (52%)	1.05 (0.94, 1.18)	
	**Unknown**	194 (4%)	102 (4%)	92 (4%)	1.03 (0.76, 1.38)	

The mean age of subjects at enrollment was 65.7±12.3 years (mean ± standard deviation [SD]), with cases being significantly older than controls (70.2±11.3 vs. 61.6±11.8 years, P<0.003).

At baseline, cases had higher rates of systemic hypertension (80% vs. 72%, OR 1.58, 95% CI 1.38–1.81, P<0.001), but less diabetes (40% vs. 46%, OR 0.79, 95% CI 0.71–0.89, P<0.001) and lower BMI (OR 0.80 per 5 BMI unit, 95% CI 0.76–0.83, P<0.001) than controls. There was no significant difference in smoking status between cases and controls.

In the multivariable model, male gender was significantly associated with risk of POAG (OR 1.64; 95% CI, 1.44–1.87; P<0.001), after adjusting for age, systemic hypertension, diabetes, and BMI. The association of POAG with these potentially confounding variables is shown in Table 2.

**Table 2:** Multivariable analysis of demographic variables and co-morbidities associated with POAG.

Effect	Point Estimate	p-value
**Sex: Male vs Female**	1.64 (1.44, 1.87)	<0.001
**Age: per year**	1.86 (1.76, 1.97)	<0.001
**Diabetes: Yes vs No**	0.75 (0.66, 0.86)	<0.001
**Hypertension: Yes vs No**	1.20 (1.03, 1.40)	0.02
**BMI: per 5 kg/m**^**2**^	0.86 (0.78, 0.94)	0.001

**Fig 1:**
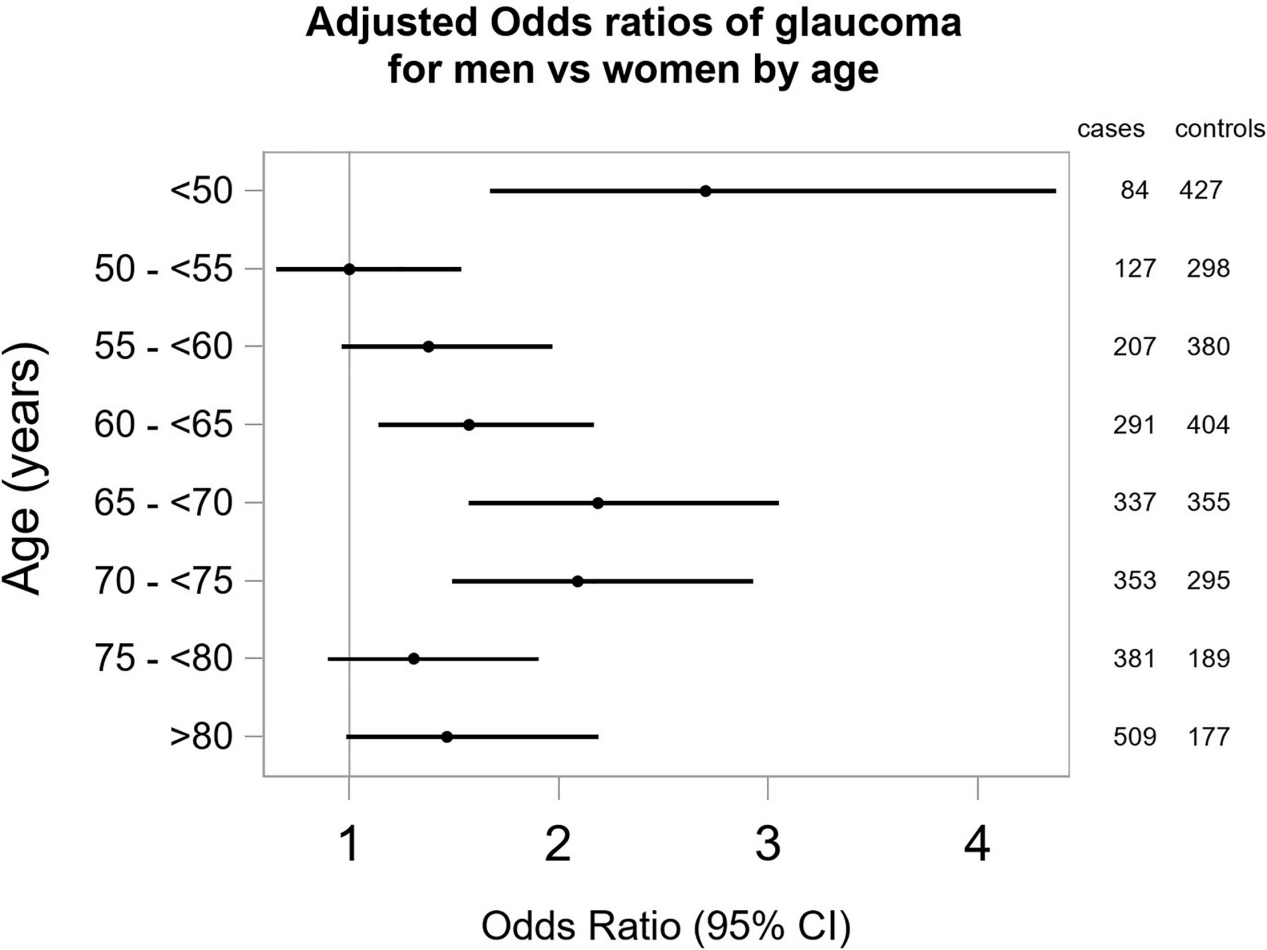
The Primary Open-Angle African American Glaucoma Genetics (POAAGG) Study: Gender differences in primary open angle glaucoma by age (per 5 years). The odds of POAG in men was as high or higher than in women across all age groups, and varied across age (p-value for interaction = 0.02). However, there was no consistent trend with increasing age, as observed highs occurred at <50 (adjusted OR 2.70 [1.67, 4.37]) and 65-<70 (2.19 [1.57, 3.05]), while observed lows occurred at 50-<55 (1.00 [0.65, 1.53]) and 75-<80 (1.31 [0.90, 1.90]).

Because of hormonal changes that occur during aging, we examined the gender-specific risk of POAG by age (per 5 years) (Fig 1). The risk of having POAG was higher for men across all age groups, particularly at age less than 50 years old (adjusted OR 2.75, 95% CI 1.70–4.45). However, at the age of 50–55 (age of menopause), men and women had almost the same risk of having POAG (adjusted OR 1.02, 95% CI 0.66–1.56).

## Discussion

In this report, we showed that males in a large African American cohort were 1.64 times more likely to have POAG than females, after adjusting for age differences, systemic diseases, and BMI. POAG cases also had more systemic hypertension, less diabetes, and lower BMI than controls.

A summary of the findings from population-based prevalence studies for POAG is displayed in Table 3. In the Framingham Eye Study [9], prevalence of definite open-angle glaucoma (OAG) was higher in men than women (2.5% vs. 1.4%), with an age-adjusted OR of 1.8. In the Rotterdam Study [10], men had a more than three times higher risk of having POAG than women (1.9% vs 0.6%), with adjusted OR of 3.6. The Los Angeles Latino Eye Study also reported a higher rate of POAG for men than for women (5.5% vs 4.4%), with adjusted OR of 1.64.

**Table 3:** Prevalence of POAG in men vs women as reported by population-based prevalence studies.

Population Based Prevalence Studies	Study Sample	Odds Ratio (95% CI) (p-value)	POAG Prevalence
**Baltimore Eye Survey[4]**	Total = 5308 African-American 45% White 55%	Age- and race- adjusted RR 1.15 (P = 0.39)^a^	2.7% in men vs 2.4% in women
**Barbados Eye Study[8,13]**	Total = 4631 Black 93% Mixed race 4%	Adjusted OR 1.66 (95% CI, 1.24–2.24)	8.3% in men vs 5.7% in women
**Framingham Eye Study[9]**	White^b^ (n = 2631)	OR 1.8 (P<0.05)	2.5% in men vs 1.4% in women
**Rotterdam Study[10]**	White^b^ (n = 6780)	OR 3.6 (P<0.05)	Higher in men in all age groups
**Blue Mountains Eye Study[7]**	White^b^ (n = 3654)	Age- adjusted OR 0.66 (95% CI, 0.45–1.00)	Slightly higher in women in all age groups
**Melbourne Visual Impairment Project[5]**	White^b^ (n = 4744)	RR 1.00 ^a^	1.8% in men vs 1.8% in women
**Projecto VER in Southern Arizona[6]**	^c^Hispanics (n = 4774)	OR 0.85 (95% CI, 0.56–1.31)	1.79% in men vs 2.1% in women
**Los Angeles Latino Study[11]**	^c^Latino (n = 6357)	Adjusted OR, 1.64 (95% CI, 1.23–2.2)	5.5% in men vs 4.4% in women
**National Health and Examination Survey (2005–2008)[3]**	Total = 5746 White 75.8% African American 10.2% Mexican American 5.6%	RR 1.26 ^a^ Estimated OR 1.32 (95% CI, 0.97–1.79)	2.4% in men vs 1.9% in women

^a^ We estimated risk ratio (RR), which for rare disease such as POAG is close to OR

^b^ Predominantly white population, with other racial groups <5%

^c^ Hispanics or Latino population only

It is of particular importance that our results are similar to POAG gender differences in among participants of the population-based Barbados Eye Study who identified as black (93%) and mixed race (4%) [8]. In this study, the prevalence of POAG was 8.3% in men and 5.7% in women [8], with an OR of 1.66 [13]. Similar to our study, the Barbados Eye Study criteria for definite OAG was based on both optic disc deterioration and VF defects. The Barbados Eye Study reported in the final model, which included age, BMI, IOP, and family history, that men were more likely to have POAG than women (OR 1.66, 95% CI 1.24–2.24).

Most of the studies that did not demonstrate a statistically significant difference by gender reported an OR > 1 for males, except for the Melbourne Study and the Blue Mountain Study, which contained predominantly white populations. For example, the Baltimore Eye Survey reported the age- and race-adjusted rate of definite POAG for men at 2.7% and for women at 2.4% (P = 0.39). A formal meta-analysis of the estimates in Table 3 is not possible because the required elements of raw data are not available from all published studies.

Several theories have been used to explain gender differences in POAG development and progression. Anatomical differences have been observed in ocular structures between genders: men have longer axial length [14], deeper anterior chamber [14], larger disc area [15], thinner retinal nerve fiber layer [16], and higher IOP [17]. Gender differences in ocular hemodynamics have also been reported [18], possibly due to the vasodilator effect of estrogen, which results in enhanced ocular blood flow [19]. Moreover, there are estrogen receptors in ciliary epithelium, suggesting estrogen could influence aqueous humor secretion and drainage [20]. Estrogen-meditated protection, including neuroprotective effects, antioxidant properties, and activation of immune system [20], could provide some protective effect to the optic nerve. If so, during and after menopause, women may have the same risk of POAG as men, due to decrease of estrogen-meditated protection. Data from other studies provide evidence of role of menopause in POAG pathogenesis. The Rotterdam Study found that early menopause was associated with higher risk of POAG[21] and the Nurses’ Health Study found that entering menopause at age 54 or greater was associated with reduced POAG risk.[22] These studies were not conducted solely in African Americans; however African American and Caucasian females do not have significant differences in age of menopause.[23,24] In order to assess the hypothesis that declining estrogen levels associated with menopause would decrease the difference in risk between men and women, we investigated gender differences across age groups by stratifying our data by age (Fig 1). The odds ratios for POAG in males compared to females varied significantly by age (p-value for interaction = 0.02), but showed no consistent trend with increasing age.

Another important theory explaining gender differences in glaucoma is based on genetic variations, including the heterogametic sex hypothesis and mitochondrial inheritance [25]. The POAAGG study’s genetic data, obtained through a genome-wide association study and whole-exome sequencing, will be used to better understand the variants underlying gender differences in POAG.

The male preponderance in cardiovascular diseases could also be partly explained by gender differences in glaucoma. Some studies have reported common risk factors between POAG and vascular disease, such as systemic hypertension and diabetes, and, possibly, obesity [13,26,27]. In our study, hypertension was positively associated with POAG, agreeing with studies such as the Rotterdam Study [26] and the Blue Mountains Eye Study [27]. Our study also showed that lower BMI was associated with POAG. Though prior studies have shown that obesity increases risk of ocular hypertension[28,29], studies on BMI and glaucoma remain more inconclusive. The Barbados Eye Study (27)and Singapore Malay Eye Study[30] also found that higher BMI decreased likelihood of POAG, though the reasons for this association were not clear. On the other hand, a hospital-based study reported that patients with higher BMI were *more* likely to have a glaucoma diagnosis [31]. BMI measurements likely do not fully account for the genetic mechanisms that influence body mass, so further research with new, more targeted body mass and adiposity measurement tools could be helpful in elucidating this association[32]. Finally, in our study, higher rates of diabetes were reported among controls, which could reflect hospital-based control recruitment from UPenn Ophthalmology Department. Results from prior studies vary: several found a protective relationship between POAG and diabetes[33], while others reported no relationship[34,35] or a positive association[36]. After controlling for systemic risk factors in the multivariable model, male gender was still significantly associated with POAG.

Our study is the largest genetic study of POAG in an African American population recruited in a single city. Every effort was made to ensure high enrollment rate to the POAAGG study [12], including community integration, outreach and in-reach screenings, and strong relationships between glaucoma specialists and participants. Although we do not have a complete ocular and systemic profile of those who declined to participate in the study (non-respondents), previous reports from the POAAGG study showed no significant difference between non-respondents versus study participants in terms of main socio-demographic parameters, including gender, although non-respondents tended to be older [37].

There are several limitations to this study. First, unlike the studies presented in Table 3, our study sample was not population-based; participants for the POAAGG study were recruited primarily from tertiary ophthalmic practices, possibly introducing selection bias. However, we believe that inclusion of major hospitals in the area and inclusion of patients from a private practice in West Philadelphia increased generalizability of the study results. [12] Second, controls for the POAAGG study were recruited during eye appointments, and therefore may be more likely to have other (non-POAG) ocular diseases. Third, cases had a higher mean age than controls in this study; we accounted for this age difference by adjusting all analyses for age and examining the gender-specific risk of POAG by age. Finally, most of the socio-demographic, systemic, and anthropometric information was self-reported and thus could be subject to recall bias. However, reports in the literature suggest that self-reporting is accurate among various ethnic groups and correlates well with medical records.[38] To address this limitation, whenever participants did not know or could not recall key data, this data was verified and/or extracted from medical records.[12]

In conclusion, this report showed that African American men had higher risk of POAG than African American women when controlling for age, systemic diseases, and anthropometrics. Further research is needed to evaluate the complex interaction of ophthalmic, genetic, and systemic variables to deepen our understanding of gender differences in POAG in African Americans.

## Supporting information

S1 File Data. (XLSX)
